# Correlation between dental arch form and OSA severity in adult patients: an observational study

**DOI:** 10.1186/s40510-023-00464-5

**Published:** 2023-05-29

**Authors:** Domenico Ciavarella, Alessandra Campobasso, Elisa Conte, Giuseppe Burlon, Laura Guida, Graziano Montaruli, Michele Cassano, Michele Laurenziello, Gaetano Illuzzi, Michele Tepedino

**Affiliations:** 1 Department of Clinical and Experimental Medicine, University of Foggia, Via Rovelli, 50, 71122 Foggia, Italy; 2 Department of Biotechnological and Applied Clinical Sciences, University of L’Aquila, L’Aquila, Italy

**Keywords:** 3D printing, Craniofacial anomalies, Obstructive sleep apnea, Morphometrics, Sleep apnea

## Abstract

**Background:**

The role of interdental widths and palatal morphology on the development of obstructive sleep apnea (OSA) has not been well investigated in adult patients yet. The aim of this paper was to assess the morphology of maxilla and mandibular dental arches on three-dimensional (3D) casts and to correlate these measurements with the severity of OSA.

**Methods:**

Sixty-four patients (8 women and 56 men, mean age 52.4) with a diagnosis of mild-to-moderate OSA were retrospectively enrolled. On each patient, home sleep apnea test and 3D dental models were collected. Apnea-hypopnea index (AHI) and oxygen desaturation index (ODI) were recorded, as well as the dental measurements including inter-molar distance, anterior and posterior widths of maxillary and mandibular arches, upper and lower arch lengths, palatal height, and palatal surface area. The respiratory and dental variables were then correlated.

**Result:**

A statistically inverse correlation was found between ODI and anterior width of lower arch, maxillary arch length, palatal height, and palatal area. AHI showed a significant inverse correlation with anterior width of mandibular arch and maxillary length.

**Conclusion:**

A significant inverse correlation between maxillary and mandibular morphology and respiratory parameters was shown in the present paper.

## Introduction

Sleep-related breathing disorders (SRBD) include a large spectrum of breathing anomalies that occur during sleep, ranging from chronic or habitual snoring to obstructive sleep apnea (OSA).

As reported by the American Academy of Sleep Medicine (AASM), OSA is the most common SRBD, and it is characterized by recurrent episodes of obstruction of the upper airways that cause oxygen desaturation [1]*.* In fact, the repetitive collapse and reopening of the airways during night induces an intermittent hypoxia and hypercapnia [2] which lead to a sleep fragmentation, loud snoring, increased respiratory effort and significant worsening of the sleep quality [3]. These conditions are also associated with a wide range of clinical symptoms both in the short and the long term, such as excessive daily sleepiness, decreased quality of life, increased risk of accidents, cardiovascular mortality and morbidity, cognitive or metabolic diseases [2, 4].

Although the pathogenesis of OSA is related to a narrow and collapsible pharynx, its pathophysiology is complex [5, 6]. In fact, a large variety of factors may influence the caliber of the upper airway during sleep, affecting the anatomic dimensions of the upper airway, the pharyngeal muscle control and the central control of respiration [7]. These factors, alone or in combination, may obstruct the passage of air in the upper airways [8], because the reduction of the muscular tone and the alteration of the respiratory control may cause a pharyngeal lumen constriction and an increase in the airway resistance which induce a partial or total obstructive events, in predisposed subjects [4].

At the same time, abnormalities of the facial skeleton may contribute to the OSA development, affecting both hard and soft tissues [9]. The most frequent alterations are reduced maxillary and mandibular dimensions, distal position of hyoid bone, long face, mandibular retroposition, narrow upper airway, increased volume of tongue and soft palate, hypertrophy of adenoids and tonsils (especially in growing children) [2, 4].

Many studies have focused on the influence of interdental width and palatal morphology on the onset of OSA in growing patients [4]. Previous literature is agreed that pediatric subjects with narrow or high-arched hard palate are predisposed to OSA and often present dental crowding or malocclusion [10, 11].

Nevertheless, despite the agreement of literature on the role of narrow arch, in adult patients the influence of the maxillary morphology and the dental widths on the development of OSA is scarce and still unclear [4], reporting contrasting results.

According to Seto et al. [12], the results of Irlandese et al*.* [4] reported that OSA patients had a narrower dental arch form compared to healthy subjects, and that the interdental widths at molar, premolar and canines level were significantly reduced, both in the maxillary and mandibular arches. Instead, although a smaller palatal volume was reported in apneic patients compared to controls, Kecik et al*.* [2] found that only the inter-molar width was significantly reduced in OSA subject, while the inter-canine distance showed no statistical differences between two groups. Moreover, Johal and Conaghan [13] did not find any significant differences in the interdental widths, even if significant differences were observed between OSA and control subjects in the palatal height measurements and in the maxillary morphology.

Therefore, although craniofacial characteristics are considered among the predisposing factors for OSA, the role of the dental arch form and palatal vault in the etiology of OSA has not been accurately described yet, and this shortcoming needs to be addressed to develop evidence-based clinical practice.

The aim of this study was to evaluate the morphology of maxillary and mandibular dental arches of adult SRBD patients and to determine any correlation of these dental dimensions with the indexes of OSA severity.

## Materials and methods

A power analysis (G*Power 3.1.9.2, Franz Faul, Universitat Kiel, Germany) revealed that to detect a large effect size of 0.4 [14] with a correlation test, considering an alpha of 0.05 and a power of 0.90, 58 subjects would be needed. A 10% was added to this calculation, leading to a final sample size of 64.

Thus, the sample consisted of 64 patients (8 women and 56 men, mean age 52.4, age range 21-79 years) that were retrospectively enrolled in the present study from patients visited at the Orthodontics and Sleep Medicine Unit of the Dental Clinic of the University of Foggia, and that referred a sleep related breathing disorder (SRBD). All the procedures of this research protocol have adhered to the Declaration of Helsinki and have been approved by the Ethics Committee of the University of Foggia (Approval no. 43/CE/2019). A written consent was signed by each patient.

### Patients criteria selection

Patients were screened in chronological order from January 2012 to January 2022 according to the following inclusion criteria: Age greater than 18 years old, body mass index (BMI) lower than 33,9 kg/m2, a diagnosis of OSA (i.e., from simple snoring to severe OSA) confirmed by HSAT.

Exclusion criteria were as follows: smoking habit, periodontitis or tooth loss, any comorbidities such as cardiovascular or pulmonary diseases, previous cervical trauma or neurological disorders.

### Models evaluation

Dental impressions of sixty-four patients were scanned with intraoral scanner TRIOS 3® (3Shape, Copenhagen, Denmark). 3D models were analyzed using Ortho Viewer® (3Shape, Copenhagen, Denmark) and Autodesk Meshmixer® (San Rafael, CA, USA) software.

**Fig. 1:**
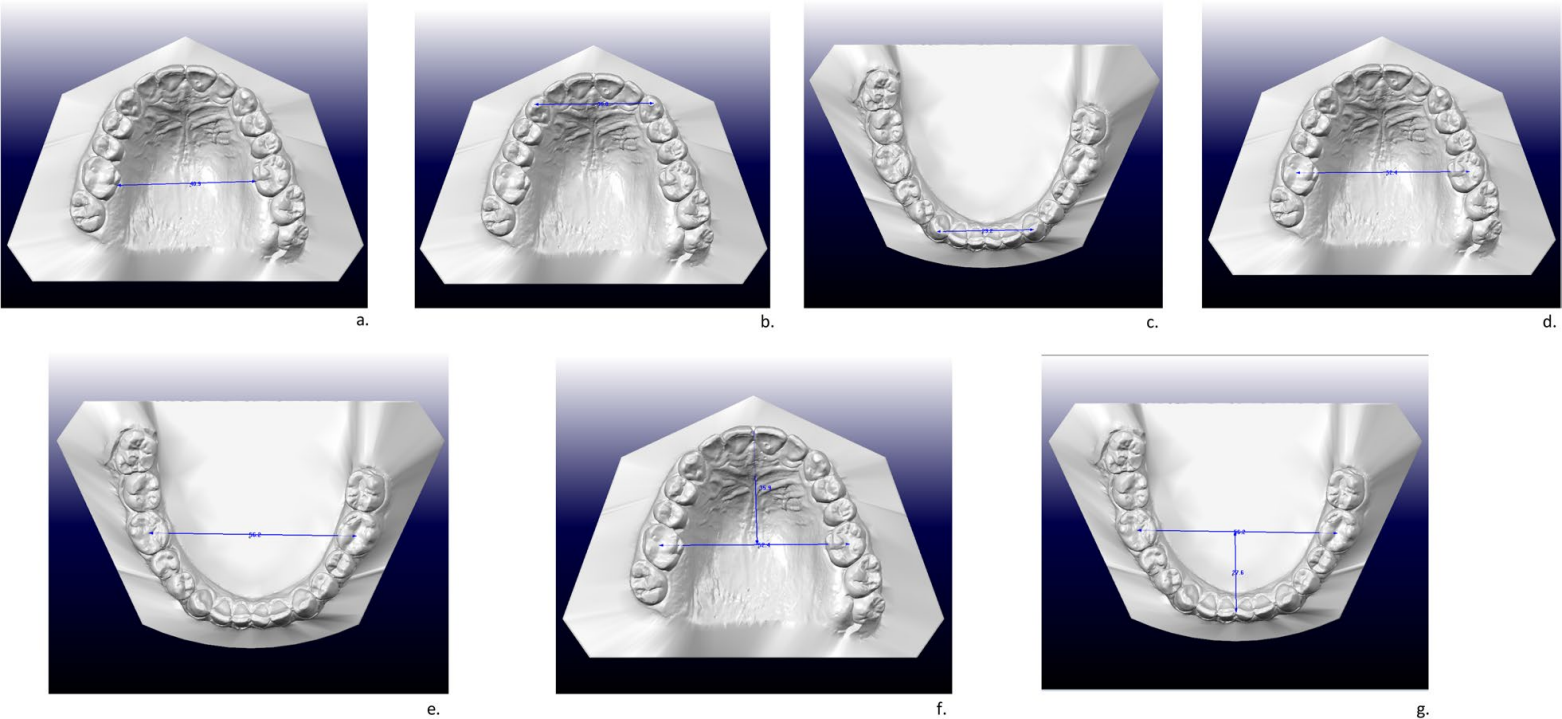
**a**. Upper inter-molar distance, **b**. Anterior width of upper arch, **c**. Anterior width of lower arch, **d**. Posterior width of upper arch, **e**. Posterior width of lower arch, **f**. Upper arch length, **g**. Lower arch length

Using the “Measurement” function on Ortho Viewer® software, the following linear measurements were recorded in the sagittal, vertical and transversal plane, as follows:

- Inter-molar distance, defined as the distance between the palatal grooves of the upper first molars with the gingiva [15] (Fig. 1a).
- Anterior arch widths, defined as the distance between the centroids of the upper and lower canines, as described by Moyers et al. [16] (Fig. 1b,c).
- Posterior arch widths, defined as the distance between the centroids of the upper and lower first molars [16] (Fig. 1d,e).
- Maxillary and mandibular arch lengths, namely the arch depth from the midpoint of the most labial points of the central incisors to the maxillary first molars at the centroid points (Figs. 1f, g).

**Fig. 2:**
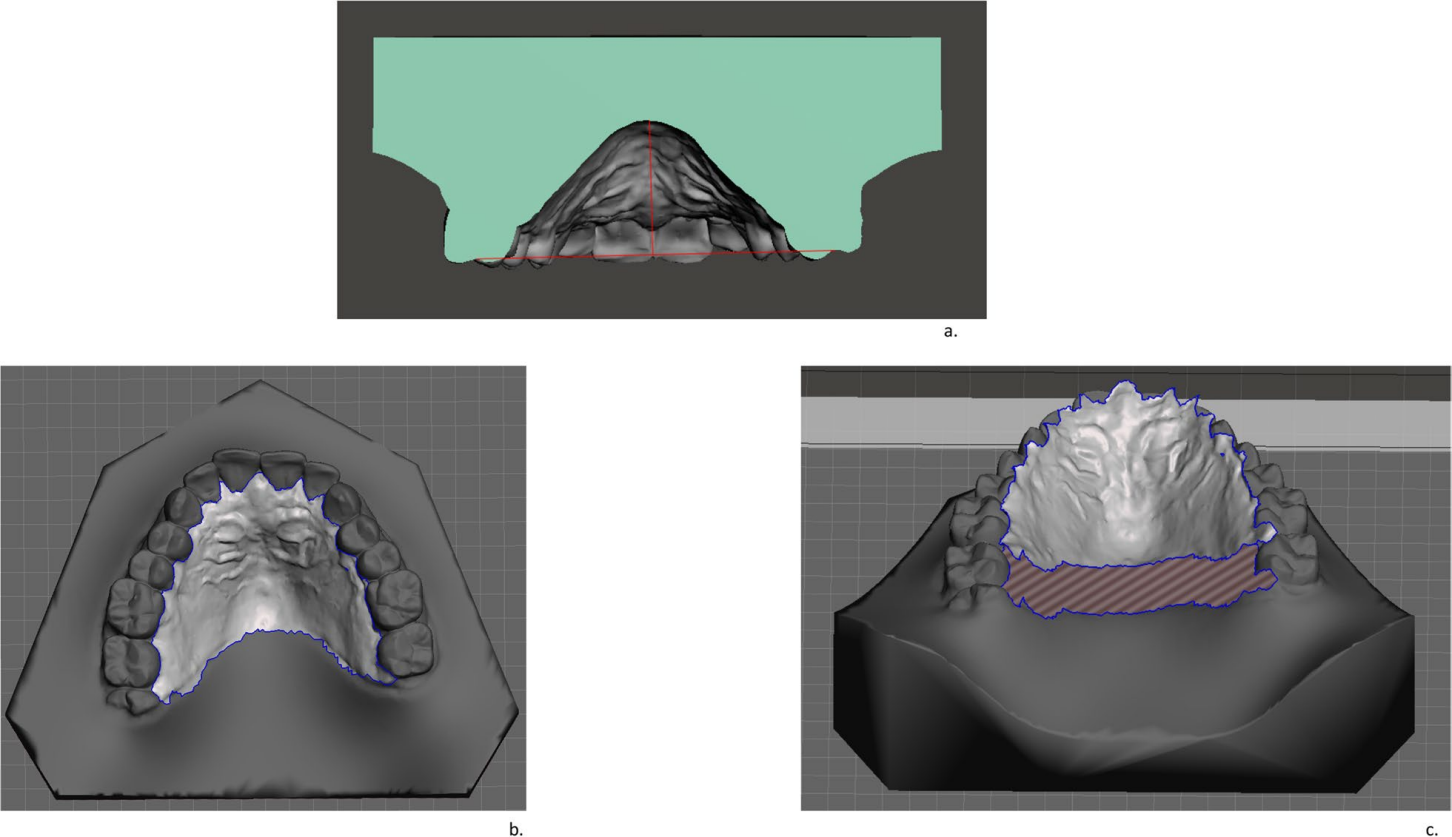
**a**. Palatal height, **b**. Palatal surface area, **c**. Palatal surface area separated from the rest of the maxilla

Using Autodesk Meshmixer®, a linear and a 3D measurements were also recorded, through "point to plane" and “select brush” function, respectively:

- Palatal height, defined by drawing a mid-sagittal plane along the median palatine raphe, perpendicular to the horizontal plane between right and left upper first molars. Considering this orientation, the intersection line of this sagittal plane in the model allowed to measure the palatal depth along the median raphe perpendicular to the inter-molar plane [15] (Fig. 2a). The maximum distance was used for analysis.
- Palatal surface area, defined as the palatal area isolated by the rest of the model and expressed in mm^2^ (Fig. 2b, c).

To estimate the method error, fifteen digital models were randomly selected and the measurements were repeated by the same operator at a one month interval. Then, the Dahlberg’s formula was used to calculate the random error between the two sets of measurements.

### HSAT analysis

Each patient received a HSAT. Although with some limitations, HSAT is considered a valuable tool for OSA assessment by the American Academy of Sleep Medicine (AASM) [17]. The registration was considered effective if the device successfully registered for more than 4 h without interruption. The data from the HSAT records were used for a manual scoring according to the AASM criteria from 2007 [18]. All subjects were evaluated for one night using a type 2 portable device (Embletta system X-100, Flaga, Reykjavik, Iceland). Overnight continuous recordings of oxygen saturation were obtained by a finger pulse oximeter. The detailed description of the evaluated parameters is shown in Table 1.

**Table 1:** Description of HSAT parameters

Apnea-hypopnea index (AHI)	The number of apnea and hypopnea events per hour of sleep. It defined the OSA severity as followed: mild, 5–14.9; moderate, 15–30; severe, > 30. An AHI of < 5 was considered normal
Oxygen desaturation index (ODI)	The number of times that oxygen desaturates by 4% per hour of sleep

### Statistical analysis

Data have been analyzed using GraphPad Prism software 6.0 (GraphPad Prism Software, San Diego, CA, USA). Data were evaluated using Shapiro–Wilk normality test with a confidence level of 95%. Mean, standard deviation (SD), standard error of mean (SEM) and upper and lower 95% confidence of mean were evaluated. Because the majority of the variables were not normally distributed, a nonparametric statistical test was used (Spearman’ rho test). To evaluate the possible effect of sex, two different tests were then performed for males and females separately. Type error was set as *p* < 0.05 for all the tests.

## Results

All sixty-four patients included in the study showed an OSA from mild to severe entity with a mean AHI of 23.98 and a mean ODI of 17.48 (Table 2). The random error was between 0.14 and 0.17 mm, with the only exception of the inter-molar width that showed a random error of 0.47 mm. The results of statistical analysis are shown in Table 3, 4, 5 and 6.

**Table 2:** Statistical analysis of data

	Palatal height	Upper inter-molar distance	Anterior width of upper arch	Posterior width of upper arch	Anterior width of lower arch	Posterior width of lower arch	Length of the upper arch	Length of the lower arch	ODI	AHI	Palatal area
Number of values	64	64	64	64	64	64	64	64	64	64	64
Mean	23.26	37.26	35.00	48.43	26.35	44.97	34.21	30.51	17.48	23.98	2131
Std. Deviation	4.223	3.344	2.860	3.649	2.707	4.416	4.158	3.841	12.92	14.84	348.5
Std. Error of Mean	0.5279	0.4180	0.3575	0.4562	0.3383	0.5520	0.5198	0.4801	1.615	1.855	43.56
Lower 95% CI of mean	22.20	36.43	34.28	47.52	25.67	43.86	33.17	29.55	14.26	20.27	2044
Upper 95% CI of mean	24.31	38.10	35.71	49.34	27.02	46.07	35.25	31.47	20.71	27.68	2218
Normality Test	No	Yes	No	Yes	No	No	Yes	No	No	No	Yes

**Table 3:** Spearman Rho test for correlation between patient’s age and OSA severity

	Age	AHI	ODI
Age		− 0.160	0.023
AHI	− 0.156		0.699
ODI	0.023	0.699

**Table 4:** Spearman's rank correlation coefficient

	Palatal height	Upper inter-molar distance	Anterior width of upper arch	Posterior width of upper arch	Anterior width of lower arch	Posterior width of lower arch	Length of the upper arch	Length of the lower arch	ODI	AHI	Palatal area
Palatal height		*− *0.014°	0.0465°	0.009°	0.105°	*− *0.026°	0.184°	*− *0.221°	*− *0.323**	*− *0.147°	0.182°
Upper inter-molar distance	*− *0.014°		0.518**	0.920**	0.187°	0.628**	*− *0.008°	0.075°	0.044°	*− *0.093°	0.234°
Anterior width of upper arch	0.047°	0.518**		0.435**	0.413**	0.398**	0.176°	0.136°	*− *0.065°	*− *0.114°	0.391**
Posterior width of upper arch	0.009°	0.920**	0.4351**		0.196°	0.615**	*− *0.002°	0.049°	0.130°	*− *0.014°	0.184°
Anterior width of lower arch	0.105°	0.187°	0.413**	0.196°		0.337**	0.491**	*− *0.004°	*− *0.323**	*− *0.419**	0.396**
Posterior width of lower arch	*− *0.026°	0.628**	0.398**	0.615**	0.337**		0.024°	0.105°	0.097°	*− *0.026°	0.310*
Length of the upper arch	0.184°	*− *0.008°	0.176°	*− *0.002°	0.491**	0.024°		0.051°	*− *0.559**	*− *0.439**	0.376**
Length of the lower arch	*− *0.221°	0.075°	0.136°	0.050°	*− *0.004°	0.105°	0.051°		0.184°	0.092°	0.075°
ODI	*− *0.323**	0.0441°	*− *0.065°	0.130°	*− *0.323**	0.097°	*− *0.559**	0.184°		0.683**	*− *0.330**
AHI	*− *0.147°	*− *0.093°	*− *0.114°	*− *0.0138°	*− *0.420**	*− *0.026°	*− *0.439**	0.092°	0.683**		*− *0.167°
Palatal area	0.182°	0.234°	0.391**	0.184°	0.396**	0.310*	0.376**	0.075°	*− *0.330**	*− *0.167°	

°ns; *p < 0.05; **p < 0.01

**Table 5:** Spearman Rho test for correlation between males and OSA severity

	Palatal height	Upper inter-molar distance	Anterior width of upper arch	Posterior width of upper arch	Anterior width of lower arch	Posterior width of lower arch	Length of the upper arch	Length of the lower arch	ODI	AHI	Palatal area
Palatal height		*− *0.026	*− *0.200	0.009	0.065	*− *0.058	0.155	*− *0.294	*− *0.374	*− *0.177	0.072
Upper inter-molar distance	*− *0.026		0.653	0.907	0.254	0.607	0.054	0.097	*− *0.035	*− *0.183	0.233
Anterior width of upper arch	*− *0.200	0.653		0.548	0.482	0.418	0.177	0.102	*− *0.168	*− *0.217	0.222
Posterior width of upper arch	0.009	0.907	0.548		0.252	0.588	0.050	0.078	0.063	*− *0.092	0.175
Anterior width of lower arch	0.065	0.254	0.482	0.252		0.359	0.422	*− *0.011	*− *0.336	*− *0.421	0.417
Posterior width of lower arch	*− *0.058	0.608	0.418	0.588	0.359		0.029	0.123	0.068	*− *0.060	0.274
Length of the upper arch	0.1545	0.054	0.177	0.050	0.422	0.029		0.028	*− *0.587	*− *0.421	0.427
Length of the lower arch	*− *0.294	0.097	0.102	0.078	*− *0.011	0.123	0.028		0.191	0.086	0.059
ODI	*− *0.374	*− *0.035	*− *0.168	0.063	*− *0.336	0.068	*− *0.587	0.191		0.672	*− *0.400
AHI	*− *0.177	*− *0.183	*− *0.217	*− *0.0916	*− *0.421	*− *0.060	*− *0.421	0.086	0.672		*− *0.224
Palatal area	0.0715	0.233	0.222	0.175	0.417	0.274	0.427	0.060	*− *0.400	*− *0.224	

Palatal height		0.848	0.139	0.945	0.636	0.671	0.254	0.02790339	0.005	0.191	0.600
Upper inter-molar distance	0.849		4.877	6.966	0.058	7.037	0.693	0.4762127	0.799	0.177	0.084
Anterior width of upper arch	0.139	4.877		1.234	1.668	0.001	0.192	0.4530857	0.217	0.108	0.100
Posterior width of upper arch	0.945	6.966	1.234		0.061	1.850	0.715	0.5701771	0.647	0.502	0.196
Anterior width of lower arch	0.636	0.058	1.668	0.061		0.007	0.0011	0.9341547	0.012	0.001	0.001
Posterior width of lower arch	0.671	7.037	0.001	1.850	0.007		0.831	0.3678089	0.619	0.661	0.041
Length of the upper arch	0.254	0.693	0.192	0.715	0.001	0.831		0.8395128	2.030	0.001	0.001
Length of the lower arch	0.028	0.476	0.453	0.570	0.934	0.368	0.840		0.158	0.529	0.664
ODI	0.005	0.799	0.217	0.647	0.012	0.619	2.030	0.158		1.456	0.002
AHI	0.191	0.177	0.108	0.5021	0.001	0.662	0.001	0.529	1.456		0.0978
Palatal area	0.600	0.084	0.100	0.196	0.001	0.041	0.001	0.664	0.002	0.099

**Table 6:** Spearman Rho test for correlation between females and OSA severity

	Palatal height	Upper inter-molar distance	Anterior width of upper arch	Posterior width of upper arch	Anterior width of lower arch	Posterior width of lower arch	Length of the upper arch	Length of the lower arch	ODI	AHI	Palatal area
Palatal height		*− *0.638	0.521	*− *0.702	0.278	*− *0.733	0.432	0.503	*− *0.232	*− *0.366	0.351
Upper inter-molar distance	*− *0.639		*− *0.366	0.985	*− *0.409	0.778	*− *0.419	*− *0.358	0.697	0.563	*− *0.420
Anterior width of upper arch	0.521	*− *0.366		*− *0.403	0.173	*− *0.195	0.220	0.312	0.128	*− *0.121	0.556
Posterior width of upper arch	*− *0.702	0.985	*− *0.403		*− *0.283	0.840	*− *0.330	*− *0.437	0.642	0.478	*− *0.449
Anterior width of lower arch	0.278	*− *0.409	0.173	*− *0.283		*− *0.035	0.888	0.006	*− *0.368	*− *0.632	0.193
Posterior width of lower arch	*− *0.733	0.778	*− *0.195	0.840	*− *0.035		*− *0.102	*− *0.609	0.317	0.107	*− *0.063
Length of the upper arch	0.432	*− *0.419	0.220	*− *0.330	0.888	*− *0.102		0.270	*− *0.489	*− *0.767	0.166
Length of the lower arch	0.503	*− *0.358	0.312	*− *0.437	0.006	*− *0.609	0.270		*− *0.047	0.075	0.008
ODI	*− *0.232	0.697	0.128	0.642	*− *0.368	0.317	*− *0.489	*− *0.047		0.827	*− *0.315
AHI	*− *0.367	0.563	*− *0.121	0.478	*− *0.632	0.107	*− *0.767	0.0745	0.827		*− *0.281
Palatal Area	0.351	*− *0.420	0.556	*− *0.449	0.193	*− *0.063	0.166	0.008	*− *0.315	*− *0.281	

Palatal height		0.089	0.185	0.0524	0.505	0.039	0.285	0.204	0.581	0.373	0.394
Upper inter-molar distance	0.089		0.372	8.639	0.314	0.023	0.301	0.384	0.055	0.146	0.300
Anterior width of upper arch	0.185	0.372		0.322	0.683	0.644	0.601	0.451	0.762	0.775	0.1521
Posterior width of upper arch	0.052	8.639	0.322		0.497	0.009	0.424	0.279	0.086	0.231	0.2641
Anterior width of lower arch	0.505	0.314	0.683	0.497		0.935	0.003	0.989	0.370	0.093	0.646
Posterior width of lower arch	0.039	0.023	0.644	0.009	0.935		0.809	0.109	0.444	0.801	0.882
Length of the upper arch	0.285	0.3017	0.601	0.424	0.003	0.809		0.517	0.219	0.026	0.694
Length of the lower arch	0.204	0.384	0.451	0.279	0.989	0.109	0.517		0.912	0.861	0.986
ODI	0.581	0.055	0.762	0.086	0.370	0.444	0.219	0.911		0.0112	0.448
AHI	0.373	0.146	0.775	0.231	0.093	0.801	0.026	0.860	0.011		0.500
Palatal area	0.394	0.300	0.152	0.264	0.646	0.882	0.694	0.986	0.448	0.5003	

Patient’s age was not correlated to OSA severity as expressed by the AHI and ODI indexes (Table 3); since dental arch shape is not dependent on patient’s age as well, it was decided to do not include the variable “age” into the further analysis. The Spearman analysis showed the statistical correlation between six maxillo-mandibular values and HSAT data (Table 4). A statistically significant inverse correlation was found between ODI and the morphological measurements of the palatal height (*r* = − 0,322, *p* < 0.01), of the upper length (*r* = − 0,559, *p* < 0.01), of the palatal area (*r* = − 0,330 *p* < 0.01) and of the anterior width of the mandibular arch (0,323 *p* < 0.01). A significant inverse correlation was also reported for AHI and the anterior width of the lower arch (*r* = − 0,419 *p* < 0.01) and the maxillary length (*r* = 0,439 *p* < 0.01).

When males and females were analyzed separately, the same correlation pattern was observed for male patients (Table 5). When considering female patients, the same pattern of correlation was observed, but due to the limited number of subjects only the correlation between ODI and the length of the upper arch and between ODI and AHI reached statistical significance (Table 6).

## Discussion

The role of abnormalities of craniofacial soft and hard tissues has been widely recognized in the development of OSA, and most of the studies available in literature have focused on therapeutic strategies aimed to correct or improve craniofacial structures [12].

In adult patients, continuous positive airway pressure (CPAP) is actually considered the standard non-invasive treatment for moderate-to-severe OSA [19]. However, patients’ adherence to CPAP is limited; therefore, non-CPAP therapies are frequently explored [20]. The surgical treatment of the upper airway or of the hyoid bone is also a viable treatment option, but it is an invasive method often not accepted by the patient [3]. In addition, mandibular advancement device (MAD) is a valid alternative in patients with mild-to-moderate OSA who refuse CPAP or surgical treatment [21], as recommended by AASM [1]. Recently, the Rapid Maxillary Expansion (RME) has been proposed as a treatment modality for OSA [13] and numerous systematic reviews and meta-analysis of the literature have proven the efficacy of RME to treat this disease in growing patients because the influence of the narrow palatal morphology as a predisposing factor for OSA has been assessed in pediatric patients [10, 11].

On the contrary, in adult patients, the role of dental arch form and interdental widths on OSA etiology is still unclear [4, 13]. Although some skeletal patterns may be considered as predisposing factors for OSA, dental arch form and palatal vault are not strictly related to facial form, since alveolar bone growth can be influenced by a complex compensatory mechanism [2, 13, 22].

In addition, the only few available studies that have investigated the association of arch morphology and OSA have exclusively compared the dental morphology between patients with apnea and controls, to assess any differences in size and form of the dental arches among OSA and healthy subjects [2, 4, 6, 12, 13]. Moreover, all of these articles evaluated only the upper arch, except for the study of Irlandese et al*.* [4] in which also the lower arch was considered.

As far as we know, this is the first study correlating the dental form and dimension of maxillary and mandibular arches to OSA severity. To only evaluate the influence of arch characteristics on OSA and to avoid selection bias due to obesity in the study sample, subjects with a BMI score greater than 33 were excluded. In addition, BMI was not investigated as a variable that could correlate with AHI, ODI and arch form, because previous studies have demonstrated that BMI is poorly related to the degree of OSA severity [23–25].

Only Kecik [2] and Irlandese et al. [4] used digital measurements on 3D casts, while the other studies were conducted using manual measurements on plaster models [12, 13]. In the present study, 3D models were preferred for handling and management simplicity, and to be able to evaluate the palatal area easily. The measurements evaluated by the present authors may be considered replicable and routinely used for diagnosis by every orthodontist, allowing to evaluate the dental arch dimension and morphology accurately compared to traditional manual method [26].

The present findings showed a significant inverse correlation between respiratory parameters and some dental measurements. The worsening of both AHI and ODI was associated with a significant reduction of maxillary arch length and of mandibular anterior width. A significant decrease in the palatal height and in the palatal area was also related to an increase in ODI. These findings should be explained considering that the arch constriction is a clinical predisposing factor to OSA [12]. Therefore, a reduced maxillary length and mandibular width may affect the OSA severity, promoting a low tongue position and the consequent narrowing of the back-tongue space [4].

At the same time, the palatal height itself is not a reliable indicator of maxillary contraction [12], and its role in the development of OSA is still a topic of discussion. A recent review [27] reported that palatal height does not have a significant correlation with the onset of OSA. In the present study, the inverse correlation between ODI and palatal height may be due to the reduced overall palatal surface available for the tongue, as also confirmed by the significant decrease in the palatal area reported in the current findings. Consequently, because of the smaller palatal volume, the tongue may move backward and downward, reducing the upper airway caliber during sleep and negatively influencing the OSA severity [28, 29].

According to the present results, Irlandese et al.[4] reported a significant reduction of the lower inter-canine width in OSA patients, although they also reported a significant decrease in the mandibular inter-molar width, not reported in the present study.

In the current paper, no differences in the upper interdental widths were found in the anterior and posterior area, contrasting to the results of Kecik [2] and Irlandese et al*.* [4] which demonstrated the significant decrease in inter-molar and inter-canine distances of maxillary arch in OSA group.

In the evaluation of the upper arch, Johal and Conaghan [13] reported no significant modifications in the dental transversal discrepancies, both for the anterior and posterior area, similarly to the current findings.

On the contrary, according to Seto et al*.* [12], a significant reduction of the maxillary length was demonstrated by the given results, in contrast to Johal and Conaghan [13] that did not reveal a statistical increase in the upper arch depth.

In addition to linear measurements, in the present study, the 3D analysis of the palatal surface was carried out. Among the available literature, the volumetric analysis of the palatal area was performed only by Kecik [2] which, using digital models, confirmed a significant reduction of the palatal volume in OSA patients. Moreover, in this previous study, a significant correlation between palatal morphology and pharyngeal airways was confirmed [2], and this could explain the current results in which the reduction of the palatal surface was correlated to a worsening of OSA parameters.

The clinical significance of the present study is related to OSA prevention and treatment. Since palatal height and area, maxillary sagittal and transversal length and mandibular anterior transversal length are negatively correlated with indexes of OSA severity, it is possible to infer that recognizing such pattern in the dental malocclusion could be associated with the risk of a more severe OSA.

Future study could investigate if the modification of such negative dental arch characteristics through orthodontic treatment could adjuvate SRBD treatment. Moreover, further studies could also investigate morphometric and proportions of dental arches in SRBD patients.

## Conclusions

In the present paper, authors showed that:

- An inverse correlation exists between ODI and palatal height, palatal area, maxillary length, and mandibular anterior width.
- Mandibular anterior width and maxillary length also present an inverse correlation with AHI.
- These results suggest that dental arches dimension and morphology could influence the severity of SRBD.

## Data Availability

The datasets used and/or analyzed during the current study are available from the corresponding author on reasonable request.

## References

[1] Sateia MJ (2014). International classification of sleep disorders-third edition: highlights and modifications. Chest.

[2] Kecik D (2017). Three-dimensional analyses of palatal morphology and its relation to upper airway area in obstructive sleep apnea. Angle Orthod.

[3] Ciavarella D, Campobasso A, Suriano C, Lo Muzio E, Guida L, Salcuni F (2022). A new design of mandibular advancement device (IMYS) in the treatment of obstructive sleep apnea. Cranio.

[4] Irlandese G, De Stefani A, Mezzofranco L, Milano F, Di Giosia M, Bruno G (2020). Dental arch form and interdental widths evaluation in adult Caucasian patients with obstructive sleep apnea syndrome. Cranio.

[5] Lee JJ, Sundar KM (2021). Evaluation and management of adults with obstructive sleep apnea syndrome. Lung.

[6] Banabilh SM, Samsudin AR, Suzina AH, Dinsuhaimi S (2010). Facial profile shape, malocclusion and palatal morphology in Malay obstructive sleep apnea patients. Angle Orthod.

[7] Hudgel DW (1992). The role of upper airway anatomy and physiology in obstructive sleep apnea. Clin Chest Med.

[8] Zinchuk AV, Gentry MJ, Concato J, Yaggi HK (2017). Phenotypes in obstructive sleep apnea: a definition, examples and evolution of approaches. Sleep Med Rev.

[9] Young T, Palta M, Dempsey J, Skatrud J, Weber S, Badr S (1993). The occurrence of sleep-disordered breathing among middle-aged adults. N Engl J Med.

[10] Camacho M, Chang ET, Song SA, Abdullatif J, Zaghi S, Pirelli P (2017). Rapid maxillary expansion for pediatric obstructive sleep apnea: a systematic review and meta-analysis. Laryngoscope.

[11] Sánchez-Súcar AM, Sánchez-Súcar FB, Almerich-Silla JM, Paredes-Gallardo V, Montiel-Company JM, García-Sanz V (2019). Effect of rapid maxillary expansion on sleep apnea-hypopnea syndrome in growing patients. A meta-analysis. J Clin Exp Dent.

[12] Seto BH, Gotsopoulos H, Sims MR, Cistulli PA (2001). Maxillary morphology in obstructive sleep apnoea syndrome. Eur J Orthod.

[13] Johal A, Conaghan C (2004). Maxillary morphology in obstructive sleep apnea: a cephalometric and model study. Angle Orthod.

[14] Cohen J (1992). A power primer. Psychol Bull.

[15] Blanck-Lubarsch M, Dirksen D, Feldmann R, Sauerland C, Hohoff A (2019). Children with Fetal alcohol syndrome (FAS): 3D-analysis of palatal depth and 3d-metric facial length. Int J Environ Res Public Health.

[16] Moyers REvdL FPGM, Riolo ML. Standards of human occlusal development. Center for Human Growth and Development, University of Michigan, Ann Arbor. (1976).

[17] Caples SM, Anderson WM, Calero K, Howell M, Hashmi SD (2021). Use of polysomnography and home sleep apnea tests for the longitudinal management of obstructive sleep apnea in adults: an American academy of sleep medicine clinical guidance statement. J Clin Sleep Med.

[18] Berry RB, Budhiraja R, Gottlieb DJ, Gozal D, Iber C, Kapur VK (2012). Rules for scoring respiratory events in sleep: update of the 2007 AASM manual for the scoring of sleep and associated events. Deliberations of the sleep apnea definitions task force of the American academy of sleep medicine. J Clin Sleep Med.

[19] Lorenzi-Filho G, Almeida FR, Strollo PJ (2017). Treating OSA: current and emerging therapies beyond CPAP. Respirology.

[20] Brożyna-Tkaczyk K, Myśliński W, Mosiewicz J (2021). The assessment of endothelial dysfunction among OSA patients after CPAP treatment. Medicina (Kaunas).

[21] De Meyer MMD, Vanderveken OM, De Weerdt S, Marks LAM, Carcamo BA, Chavez AM (2021). Use of mandibular advancement devices for the treatment of primary snoring with or without obstructive sleep apnea (OSA): a systematic review. Sleep Med Rev.

[22] Harvold EP, Tomer BS, Vargervik K, Chierici G (1981). Primate experiments on oral respiration. Am J Orthod.

[23] Ciavarella D, Tepedino M, Chimenti C, Troiano G, Mazzotta M, Foschino Barbaro MP (2018). Correlation between body mass index and obstructive sleep apnea severity indexes—a retrospective study. Am J Otolaryngol.

[24] Sforza E, Addati G, Cirignotta F, Lugaresi E (1994). Natural evolution of sleep apnoea syndrome: a five year longitudinal study. Eur Respir J.

[25] Pendlebury ST, Pépin JL, Veale D, Lévy P (1997). Natural evolution of moderate sleep apnoea syndrome: significant progression over a mean of 17 months. Thorax.

[26] Lo Giudice A, Ronsivalle V, Grippaudo C, Lucchese A, Muraglie S, Lagravère MO (2020). One step before 3D printing-evaluation of imaging software accuracy for 3-dimensional analysis of the mandible: a comparative study using a surface-to-surface matching technique. Materials (Basel).

[27] Kang J-H, Kim HJ, Song SI (2022). Obstructive sleep apnea and anatomical structures of the nasomaxillary complex in adolescents. PLoS One.

[28] Ciavarella D, Lo Russo L, Mastrovincenzo M, Padalino S, Montaruli G, Giannatempo G (2014). Cephalometric evaluation of tongue position and airway remodelling in children treated with swallowing occlusal contact intercept appliance (S.O.C.I.A.). Int J Pediatr Otorhinolaryngol..

[29] Tepedino M, Esposito R, Montaruli G, Monaco A, Chimenti C, Ciavarella D (2022). Changes in hyoid bone and tongue position in class I subjects after orthodontic treatment with rapid palatal expander. Cranio.

